# The effect of the 2019 coronavirus disease outbreak on social relationships: A cross-sectional study in Jordan

**DOI:** 10.1177/0020764020966631

**Published:** 2020-10-25

**Authors:** Abdallah Y Naser, Hadeel T Al-Hadithi, Eman Zmaily Dahmash, Hassan Alwafi, Salwan Salah Alwan, Zainab Ali Abdullah

**Affiliations:** 1 Faculty of Pharmacy, Isra University, Amman, Jordan; 2 College of Medicine, Umm Alqura University, Mecca, Saudi Arabia

**Keywords:** COVID-1, Jordan, relationships, social

## Abstract

**Background:**

Social relationships refer to the existing associations between family members, friends, neighbours, co-workers, and other associates. Due to the COVID-19 pandemic, social distancing has been imposed by the curfew program in Jordan.

**Aim:**

To evaluate the effects of social distancing on the social relationships of the Jordanian population.

**Methods:**

A cross-sectional study using an online survey was conducted in Jordan between the 6th and the 30th of May, 2020. Our questionnaire was constructed to explore the population’s perception of the quarantine period, how it is affecting their relationship with others, and the characteristics of their social relationships and communication with various population categories, including family members and work colleagues. Multiple linear regression was used to identify predictors of better social relationships and communication.

**Results:**

A total of 4,301 participants were involved in this study. The average score of the influence of the COVID-19 pandemic on social relationships among the whole study population was 5.68 (SD: 2.33) out of 10 (equal to 56.8%), which indicates the marginal strength of the social relationships. Around 31.6% of the participants reported that their social relationships were affected to a high degree by the COVID-19 pandemic. Participants who were aged 36–45 were positively affected in terms of their social relationships during the COVID-19 pandemic.

**Conclusion:**

The COVID-19 pandemic is negatively affecting social relationships, which could ultimately lead to negative health implications. Decision-makers are advised to provide educational campaigns that improve the sociological health of the general population.

## Introduction

Social relationships refer to existing associations between family members, friends, neighbours, co-workers, and other associates. The quality of social relationships is impacted by positive aspects such as emotional support from others, and negative aspects such as conflict and stress. Social relationship scientists often emphasise that comfortable, relaxed and easy social relationships are important in a person’s life and have a great impact on health, affecting their behavioural, psychosocial, and physiological states (Umberson & Montez, 2010). Factors associated with adverse health outcomes, including inflammatory biomarkers, impaired immune function, and even mortality, are reported to be linked to social relationships of poor quality, a low level of involvement, and a low quantity of social connections (Kiecolt-Glaser et al., 2002; Robles & Kiecolt-Glaser, 2003). Social connections have been extensively investigated, with researchers confidently agreeing on the importance of social relationships and their impact on an individual’s wellbeing (Baumeister & Leary, 1995).

The COVID-19 pandemic, also identified by the WHO as the coronavirus pandemic, causes severe acute respiratory syndrome coronavirus 2 (SARS-CoV-2). The outbreak was first reported in Wuhan, China, in December 2019 (Huang et al., 2020). The WHO declared the outbreak a pandemic on January 30th, 2020 (World Health Organization, 2020b). To date, confirmed coronavirus cases have exceeded 19,187,900, and have been reported in more than 90 countries and territories, resulting in more than 716,000 deaths (World Health Organization, 2020a).

Reports from the Centre for Disease Control and Prevention and data from published epidemiology and virology studies revealed evidence that COVID-19 is principally spread through direct exposure to the respiratory droplets of symptomatic or even asymptomatic patients, or by contact with contaminated objects and surfaces, causing a wide range of clinical and radiological symptoms (Center for Disease Control and Prevention, 2020b; Shabrawishi et al., 2020). The WHO has declared that people could be more contagious at the time of symptom onset than in subsequent stages of the disease (Center for Disease Control and Prevention, 2020a).

To reduce or even eradicate contact between people in public spaces, Jordan and many other countries worldwide implemented a curfew that came into effect on March 15th, 2020. The curfew aimed to battle the coronavirus outbreak through the application of certain regulations. Except in the case of emergencies, civilians or other unauthorised persons were forbidden to leave home and had to stay off the streets. Places of public assembly were closed. The stringent social distancing measures that were implemented in Jordan in early June 2020 remain in many sectors, particularly in education, social gatherings, sports clubs, and others. While such measures are intended to protect people from the infection, a key and unintended consequence of such instructions could be an increase in stress, loneliness and domestic violence among the whole population (Algunmeeyn et al., 2020; Piquero et al., 2020). A recent study conducted in Jordan on more than 4,000 participants found that the COVID-19 pandemic is negatively affecting the mental health of the Jordanian population, causing anxiety and depression in a considerable proportion of the population (Naser et al., 2020). Social relationships and connections enable individuals to regulate their emotions, cope with stress, and remain resilient during stressful situations. Conversely, loneliness and social isolation aggravate stress and often result in negative effects on mental, cardiovascular, and immune health (Hawkley & Cacioppo, 2010). Recently, the WHO announced that social disconnection is a major public health challenge. Interestingly, the worldwide battle to fight against the COVID-19 pandemic was based on social distancing (Courtet et al., 2020). Therefore, individuals are now facing the possibility of different forms of social isolation (Claridge, 2020). Therefore, the purpose of the present study is to evaluate the effects of social distancing imposed by the curfew program in Jordan on the social relationships of the Jordanian population.

## Methodology

### Study design and study population

A cross-sectional study using an online survey was conducted in Jordan between the 6th and the 30th of May 2020. The questionnaire instrument was constructed based on broad literature reviews on the COVID-19 pandemic and the social response to health pandemics (Center for Disease Control and Prevention, 2020a; Davis et al., 2015). The questionnaire tool was reviewed to evaluate the suitability, relevancy, simplicity, and adequacy of the questions. The questionnaire comprised 16 items, including demographic characteristics, adherence to precautionary measures, perceptions of the quarantine period and how it is affecting their relationship with others, and the characteristics of social relationships and communication with various population categories, including family members and work colleagues. The questionnaire was divided into two main sections. The first section comprised eight items on the participants’ demographic characteristics, general questions on adherence to COVID-19 precautionary measures, and perceptions of the quarantine period and how it is affecting individuals’ relationships with others. The second section included eight items that asked the participants about the nature and characteristics of their social communication with family and with colleagues at work during the COVID-19 pandemic and the resultant lockdown in the country. Five items (questions 2–6 in Table 2) in the second section were given a score from 0 to 2, where 0 represents weaker social relationships and communication, and 2 represents better social relationships and communication during the pandemic lockdown. A score of 1 was given to participants who reported that their social relationships did not change during the pandemic lockdown, resulting in a total maximum score of 10 for this section, which measures the influence of the COVID-19 pandemic and the lockdown on social relationships. The total score can be interpreted based on the mid-point of the highest possible score of the scale (which is equal to 5). The higher the total score, the better the quality of social relationships during the pandemic.

### Sampling strategy

A convenience sampling technique was employed where eligible participants were invited to participate in the study through social media (Facebook and WhatsApp). All participants were invited to participate in the study voluntarily and were thus considered exempt from written informed consent. The study aims and objectives were explained in the cover letter of the questionnaire tool.

The inclusion criteria for the study were: (a) individuals aged 18 years and above and currently living in Jordan. Participants were excluded if they were: (a) below 18 years of age, and (b) unable to understand the Arabic language (which was highlighted in the cover letter).

### Sample size

Using a confidence interval of 95%, a standard deviation of 0.5, and a margin of error of 5%, the minimum required sample size was 385 participants.

### Statistical analysis

Data were analysed using SPSS software, version 25 (IBM Corp, Armonk, NY, USA). The Kolmogorov–Smirnov and Shapiro Wilk tests were used to check the normality of the data based on their results, which provided the normality of the participants’ scores for the main outcome. Continuous variables were reported as mean (± standard deviation [SD]). Categorical variables were reported as frequencies and percentages. Participants’ scores (representing the influence of the pandemic on their social relationship and communication with others) were interpreted on a continuous scale based on the scale midpoint, where scores above the midpoint represented better social relationships and communication. A Student *t*-test and a one-way ANOVA test were used as appropriate to compare the mean scores between different demographic groups. Additionally, significant predictors of better social relationships and communication were determined using multiple linear regression analysis. A confidence interval of 95% (*p* < .05) was applied to represent the statistical significance of the results, and the level of significance was predetermined as 5%.

## Ethical considerations

This study was approved by the Research Ethics Committee at the Faculty of Pharmacy at Isra University, Amman, Jordan (PH – 2020 – 10). As participation in the study was voluntary, the research ethics committee approved the consent waiver.

## Results

### Participants’ characteristics

A total of 4,301 individual participated in this study. Most of them (36.4%, *n* = 1,523) were aged 19–25 years. Around 60.4% (*n* = 2,598) were females, and the majority (66.6%, *n* = 2,787) had secondary school education or lower. The majority of the participants (62.9%, *n* = 2,707) reported that they adhered to the precautionary measures against the coronavirus. When the participants were asked about the quality of their social relationships after the spread of COVID-19, 59.6% (*n* = 2,563) reported that they had become weaker. Also, 31.2% (*n* = 1,300) reported that the atmosphere in their family had become more strained. When the participants were asked whether they had developed certain hobbies during the quarantine period, only 36.5% (*n* = 1,569) responded that they had (Table 1).

**Table 1.** Participants’ demographics and responses to practices during COVID-19.

Demographics	Frequency (%)
Age (*n* = 4,185)	
18 years and below	174 (4.2)
19–25 years	1,523 (36.4)
26–35 years	1,162 (27.8)
36–45 years	620 (14.8)
46 years and above	706 (16.9)
Gender	
Female	2,598 (60.4)
Education level (*n* = 4,184)	
Illiterate	98 (2.3)
Secondary school or lower	2,787 (66.6)
Bachelor degree	814 (19.5)
Higher education	485 (11.6)
Do you adhere to the precautionary measures against the Corona virus?	
Yes	2,707 (62.9)
After the spread of the Coronavirus, social relations have become	
Weaker	2,563 (59.6)
How the family become during the quarantine period? (*n* = 4,170)	
More nervous	1,300 (31.2)
More considerate	906 (21.7)
Did not change	1,964 (47.1)
How did you see the quarantine period? (*n* = 4,171)	
A period of boredom	1,995 (47.8)
A period of rest and calm	1,546 (37.1)
Other than that	630 (15.1)
Have you developed certain hobbies during the quarantine period?	
Yes	1,569 (36.5)

### The effect of the COVID-19 pandemic on social relationships

The average score of the influence of the COVID-19 pandemic on social relationships among the whole study population was 5.68 (SD: 2.33) out of 10 (equals to 56.8%), which indicates a marginal social relationship. Around 30.3% (*n* = 1,303) of the participants had a total score below 5 (the midline), which indicates the negative impact of COVID-19 on individuals’ attitudes towards social relationships and communication with others.

Around 31.6% of the participants reported that their social relationships were affected to a high degree by the COVID-19 pandemic, 71.4% of the participants reported that they are spending more time with family during the COVID-19 lockdown period and only 26.2% reported that they have become more connected to their colleagues during the lockdown period (Table 2).

**Table 2.** Participants’ responses regarding the effects of COVID-19 pandemic on social relationships.

Variable	Low %	Moderate %	High %
1. Social relationships were affected, due to the corona virus, to which degree?	19.8	48.6	31.6
	Weaker %	Did not change %	Better %
2. How did the relationship of the parents with each other become during the quarantine period?	7.9	60.0	32.1
3. How did the relationship between children become between each other during the quarantine period?	7.3	52.4	40.3
4. How the relationship of parents with children became during the quarantine period?	6.6	53.1	40.3
5. How did the relationship of colleagues with each other in the quarantine period?	25.4	58.9	15.7
6. Do you think, in your view, that the Corona virus affected in a way that is?	37.8	27.4	34.8
	No %	Somehow %	Yes %
7. Are you finding more time to sit with your family after the spread of Corona virus?	7.8	20.8	71.4
8. Have you become more connected to your colleagues after the spread of the Corona virus?	30.0	43.8	26.2

The mean score differed significantly across different age groups (*p* = .014); age is positively associated with having a better mean score, which indicates better social relationships during the COVID-19 pandemic (Table 3).

**Table 3.** Participants’ mean score stratified by demographic characteristics.

Demographics	Mean	Standard deviation	*p*-Value
Age			
18 years and below	5.36	2.29	.014*
19–25 years	5.61	2.30	
26–35 years	5.79	2.31	
36–45 years	5.89	2.28	
46 years and above	5.60	2.44	
Gender			
Male	5.77	2.35	.368
Female	5.64	2.31	
Education level			
Illiterate	5.47	2.60	.354
Secondary school or lower	5.57	2.33	
Bachelor degree	5.72	2.31	
Higher education	5.68	2.32	

* *p* < 0.05.

### Predictors of the effect of COVID-19 on social relationships

Using simple linear regression, we found that participants who were aged 26–35 and 36–45, and participants who have a secondary school education, lower education, or higher education were more likely to have better social relationships during the COVID-19 pandemic. Using multiple linear regression, we found that participants who were aged 36–45 were positively affected in terms of their social relationships during the COVID-19 pandemic (Table 4).

**Table 4.** Predictors of the effect of COVID-19 on social relationships.

Variable	Simple linear regression			Multiple linear regression §		
	B	SE	β	B	SE	β
*Demographic data*						
Age						
19–25 years	0.251	0.186	.052	0.183	0.207	.038
26–35 years	0.423	0.189	.081*	0.378	0.209	.073
36–45 years	0.523	0.200	.080**	0.487	0.218	.074*
46 years and above	0.236	0.197	.038	0.187	0.218	.030
Gender						
Male	0.124	0.074	.026	0.121	0.075	.025
Education level						
Secondary school or lower	0.100	0.258	.014*	0.192	0.264	.026
Bachelor degree	0.269	0.239	.054	0.271	0.241	.055
Higher education	0.220	0.249	.037*	0.159	0.253	.027
Constant				5.137	0.306	
Adjusted *R*^2^						.002
*P*-Value						.023

*Note*. §: Includes age, gender and education level. B: The average change in the dependent variable associated with a 1-unit change in the independent variable, statistically controlled for the other independent variables. SE: the standard deviation of its sampling distribution or an estimate of that standard deviation; *β*: A statistical measure that compares the strength of the effect of each independent variable to the dependent variable.

* *p* < .05. **p < .01.

## Discussion

Personal relationships among families and friends have been reshaped during the COVID-19 pandemic. The lockdown has forced family members to live closer together, whereas others, such as friends and extended family members, have been further apart from each other. However, due to social distancing precautions, people have suffered from isolation from friends and the community (Al-Tammemi, 2020; Brooks et al., 2020; Liu, 2020). Therefore, this study aimed to identify the effects of the COVID-19 curfew system on social relationships in Jordan. The findings revealed that more than 62.0% of respondents adhered to precautionary measures against COVID-19, which included social distancing and hygiene practices. Although this is not a high percentage, it is to be expected, as 36.4% of our participants were within the age group of 16–25 years. Members of this particular age group usually find it difficult to follow social distancing and hygiene practices (Al-Tammemi, 2020; Liu, 2020).

Since cultural status in Jordan relies on a strong commitment to collective groups such as family and tribe, social distancing could have a negative impact on individuals. However, in the absence of a vaccine, social distancing has been an essential measure for slowing the pandemic. Nevertheless, social distancing conflicts with the human need to connect with others (Bavel et al., 2020). The findings of our study revealed that 59.6% of respondents believe that social relationships have become weaker during the curfew, and more than 30.0% believe that family relations have become strained, whereas 47.8% reported that the lockdown period was a source of boredom. Such findings are in line with the findings of several other research studies, which concluded that because of the lockdown, several normal activities and services have been restricted, including public gatherings, fitness centres, cinemas, retail shops, coffee shops and restaurants. As a result of the lockdown, schools and universities closed and emergency remote learning commenced to enable students to complete their academic year. Furthermore, the workforce, in general, transferred to working from home, and going out in public was extremely limited (Al-Tammemi, 2020). Such situations could lead to feelings of boredom and loneliness, which increases tension. With the cancellation of social gatherings, the inability to go to cafes, restaurants, or shopping, and with friends and family being hesitant to get together for fear of infection with COVID-19, the level of boredom and tension will escalate. Additionally, uncertainty adds to the situation where people are unaware of how long this pandemic will go on for and if ‘physical distancing’ is going to become the new normal (Claridge, 2020). Previous research found an association between boredom and anxiety or stress (Chao et al., 2020), and hence the state of boredom is something that needs to be addressed. Another study showed that quarantine and reduced social and physical contact during the COVID-19 pandemic has caused a tangible effect on boredom (Brooks et al., 2020). Only 36.5% of our participants reported that they developed new hobbies during the lockdown period, which could be attributed to the level of boredom, uncertainty and confusion. This data was collected during the initial phases of the pandemic in Jordan and people feared for the future. Ultimately, this correlates with their ability to focus and find new hobbies. Eastwood and colleagues defined boredom as ‘the aversive state of being unable to engage in satisfying activity’ (Eastwood et al., 2012). Hence, our results are justified.

Our research has further highlighted the impact of the COVID-19 pandemic on social relationships. Around 80.2% of participants reported that their social relationships were highly or moderately affected by the pandemic. Notably, the relationships between parents and children were either enhanced or not affected; hence, our results support the positive impact of the pandemic on social relationships among immediate family members. This proximity of immediate family members because of the lockdown in our study had a tangible positive impact on social relationships. Such results do not corroborate the findings of other studies, which report that forced proximity of immediate family members is a risk factor for aggression (Ellemers & Jetten, 2013; Greenaway et al., 2014). A study on the effect of COVID-19 in Asia reported that forced proximity increased the incidence of domestic violence (Owen, 2020). During the lockdown period, families were able to communicate with family members and support each other. Furthermore, in the Middle East, families had faith and strong religious beliefs, which may have contributed to their acceptance of consequences and hence their social relationship was either not affected or enhanced. The findings of this study also revealed that social relationships among siblings and with their parents did not change, or even improved. Such results are in agreement with the literature where research showed that children’s adjustment is largely dependent on the general climate and relationships (Prime et al., 2020). Several research papers reported that some families are more vulnerable in response to disasters than others, particularly those with a low income, while other families showed resilience and were able to survive through disasters (Calhoun & Tedeschi, 2014). Hence, good family relationships have demonstrated the ability to support the child in coping with trauma (Masten, 2016). In the study, 71.4% of the participants reported that they have been able to spend more time with their family members after the spread of the coronavirus. This enhanced communication among family members and strengthened the resilience of the family, according to research results (Prime et al., 2020).

This study provides further support for the notion that the social relations of friends and colleagues were negatively affected by the pandemic, where 37.8% of participants reported that these social relationships became weaker. Such results are expected as there has been a physical distance between friends and colleagues. Also, this strongly relates to responses about becoming more connected to one’s colleagues after the spread of the coronavirus, where 30.0% of participants could not communicate well and 43.8% reported that there has been some degree of connection. During the lockdown, friends and colleagues were able to connect through social media, which allowed people who are quarantined to communicate directly with their colleagues, thus reducing feelings of loneliness (Brooks et al., 2020). Indeed, other studies have reported that online interactions can also foster a sense of connection. In general, receiving and giving support online can enhance well-being (Dore et al., 2017). Other studies reported that the use of social media may not contribute to the individual’s social connection (Helliwell & Huang, 2013). Hence, social distance is replaced by physical distance, implying that there is still a social relationship among individuals, even if they are physically separated (Bavel et al., 2020).

Participants’ scores were further stratified by demographics, and findings revealed that there is a statistically significant difference regarding the impact of COVID-19 on social relationships according to age. Apart from the group above the age of 45 years old, the younger the age, the greater the effect of COVID-19 on social relationships. The age group of 18 years or below showed the lowest score, indicating a negative effect on social relationships. This association could be expected as this age group suffered from the lockdown and were deprived of their schools, friends and all social activities. The other end was pertinent to individuals aged 46 years and above. They scored 5.6 (SD: 2.44), indicating a lower number of social relationships when compared to younger groups. This is possible because this group might face greater family and job-related concerns, and fears of becoming infected. Unfortunately, there is no reported data to date on social relationships associated with the current COVID-19 pandemic. A study reported that older adults, particularly those at high risk of severe symptoms from the infection, are also highly prone to isolation (Bavel et al., 2020; Liu, 2020).

This study also found that the quality of social relationships was enhanced with the increase of age from 19 to 45 years. However, multiple linear regression showed that individuals within the age group of 36–45 were more likely to have better social relationships during the COVID-19 pandemic. This could be attributed to the ability of this age group to enhance social relationships through online and social media communication. Overall, the score for all age groups was low, highlighting the overall, negative impact of the COVID-19 on the social life of individuals.

Our results showed that education level, particularly those with higher education and lower educational level, was positively related to social relationships. People with a higher education scored better on the quality of social relationships than those with a bachelor’s degree. Highly educated individuals are probably busy with work and frequent travel. Therefore, during the lockdown, most individuals have been working from home and may have more time to spend with family members, thus increasing their enjoyment of social relationships.

## Strengths and limitations

To the best of our knowledge, this is the first and largest (4,301 participants) study in the Middle East that investigated the association between the COVID-19 pandemic and social relationships. The study has several strengths. Firstly, this is the first study in Middle Eastern Arabic-speaking countries to investigate social relationships during COVID-19. Secondly, the study addresses a critical topic as the impact of social relationships may affect health. However, there were some limitations encountered in this study. Initially, the study design itself, a cross-sectional survey design, limited our ability to identify causality between study variables. There are limited studies that have explored social relationships during the COVID-19 pandemic worldwide and in the Middle East specifically, which limited our ability to compare our findings with Arabic-speaking countries of a similar culture. Furthermore, in this study, we used a quantitative methodology with pre-set responses, which might not have allowed individuals’ views to provide varied but useful qualitative information. We were not able to estimate the response rate for our questionnaire study, which might lead to nonresponse bias, as we could not demonstrate how well the sample drawn from the population of interest, therefore, the findings should be interpreted carefully. Finally, we utilised an online survey for data collection, and hence it is possible that we missed some of the targeted population.

## Conclusion

The COVID-19 pandemic is negatively affecting social relationships, which could ultimately lead to negative health implications. Decision-makers are advised to provide educational campaigns that improve the sociological health of the general population and informing them about contemporary alternative communication media (telecommunication). This is expected to lead to better social relationships and communication across the whole population, enabling people to cope better with the pandemic and to maintain societal well-being and productivity.
